# Correction: Association of avian biodiversity and West Nile Virus circulation in *Culex* mosquitoes in Emilia-Romagna, Italy

**DOI:** 10.1371/journal.pntd.0014400

**Published:** 2026-06-02

**Authors:** The PLOS Neglected Tropical Diseases Staff

There are errors in the author affiliations. The correct affiliations are as follows:

Yiran Wang ^1,2^, Mattia Calzolari^3^, Gianpiero Calvi^4^, Victoria M. Cox^1^, Paola Angelini^5^, Michele Dottori^3^, William Wint^6^, Sally Jahn^1^, Giovanni Marini^7^, Ilaria Dorigatti^1^

**1** MRC Centre for Global Infectious Disease Analysis, School of Public Health, Imperial College London, London, United Kingdom, **2** Grantham Research Institute on Climate Change and the Environment, Imperial College London, London, United Kingdom, **3** Istituto Zooprofilattico Sperimentale della Lombardia e dell’Emilia Romagna “Bruno Ubertini”, Brescia, Italy, **4** Studio Pteryx, Basiano, Italy, **5** Settore Prevenzione collettiva e Sanità pubblica, Regione Emilia-Romagna, Bologna, Italy, **6** Environmental Research Group Oxford, Oxford, United Kingdom, **7** Research and Innovation Centre, Fondazione Edmund Mach, San Michele all’Adige Trento, Italy.

The publisher apologizes for the error.

## References

[1] Wang Y, Calzolari M, Calvi G, Cox VM, Angelini P, Dottori M, et al. Association of avian biodiversity and West Nile Virus circulation in *Culex* mosquitoes in Emilia-Romagna, Italy. PLoS Negl Trop Dis. 2026;20(3):e0014076. doi: 10.1371/journal.pntd.0014076 41790846 PMC12978567

