# Supplementary material for: Association of avian biodiversity and West Nile Virus circulation in Culex mosquitoes in Emilia-Romagna, Italy
Source: PLoS Negl Trop Dis. 2026 Mar 6;20(3):e0014076. doi: 10.1371/journal.pntd.0014076 (PMC12978567; doi:10.1371/journal.pntd.0014076)
Supplement: S1 Text — (DOCX) [file pntd.0014076.s001.docx]

**S1 Text. Equations and Parameter Details of Biodiversity Indicators in the Farmland Bird Index Data Analysis**

1. Shannon’s Diversity Index ($H$) [1]:

$$\begin{aligned} H=-\sum P_{i}\times\ln P_{i}\#\left( S1 \right) \end{aligned}$$

Where $P_{i}$ is the relative abundance of species $i$. A larger value of $H$ suggests higher diversity.

1. Simpson’s Diversity Index ($1-D$) [2]:

$$\begin{aligned} 1-D=1-\sum{P_{i}}^{2}\#\left( S2 \right) \end{aligned}$$

Where $P_{i}$ is the proportional abundance of species $i$. $D$ represents the probability that two randomly selected individuals belong to the same species. A higher value of $1-D$ indicates greater diversity.

1. Chao2 Index ($\hat{S}_{Chao2}$) [3]:

$$\begin{aligned} \hat{S}_{Chao2}=\left\{ \begin{aligned} S_{obs}+\frac{\left[ \frac{T-1}{T} \right]Q_{1}^{2}}{2Q_{2}}, if Q_{2}>0 \\ S_{obs}+\frac{\left[ \frac{T-1}{T} \right]Q_{1}\left( Q_{1}-1 \right)}{2}, if Q_{2}=0 \end{aligned} \right.\#\left( S3 \right) \end{aligned}$$

Where S_obs_ is number of observed species, T is number of samples, Q_1_ is number of species observed only once, and Q_2_ is number of species observed only twice.

**References**

1. Shannon CE. A Mathematical Theory of Communication. The Bell System Technical Journal. 1948;27:379-423.
2. Simpson EH. Measurement of Diversity. Nature. 1949;163:688.
3. Chao A. Estimating the Population-Size for Capture Recapture Data with Unequal Catchability. Biometrics. 1987;43(4):783-91. doi: 10.2307/2531532. PubMed PMID: WOS:A1987L357600004.
