# Supplementary material for: Association of avian biodiversity and West Nile Virus circulation in Culex mosquitoes in Emilia-Romagna, Italy
Source: PLoS Negl Trop Dis. 2026 Mar 6;20(3):e0014076. doi: 10.1371/journal.pntd.0014076 (PMC12978567; doi:10.1371/journal.pntd.0014076)
Supplement: S2 Text — (DOCX) [file pntd.0014076.s002.docx]

**S2 Text. Sensitivity analysis for Farmland Bird Index data**

In a sensitivity analysis, we included only bird observation records from the 68 mosquito traps that were active throughout each surveillance season from 2013 to 2023, excluding the 9 relocated traps. As in the main analysis, the smallest number of observations across WNV detection frequency groups was below 20 (Fig Aa), and the two rarefaction approaches described in the main text were applied.

**
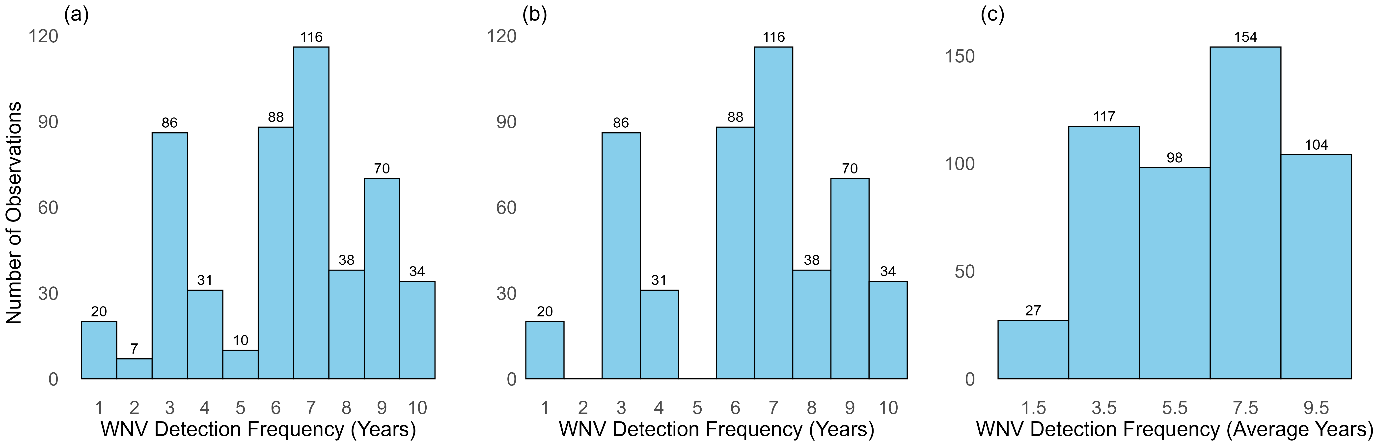
Fig A.** **Number of bird observations categorized by WNV detection frequency, based on the number of years with at least one WNV-positive mosquito pool. (a) Classification using the original group definitions.** (b) Classification after excluding groups with fewer than 20 observations. (c) Classification with the original two consecutive groups (as shown in panel a) combined.

Table A summarised the Shannon’s, Simpson’s, and Chao2 indexes obtained using the Exclusion-based Rarefaction approach based on 20 subsamples (Fig Ab). Linear regression results indicated that both rarefied Shannon’s and Simpson’s diversity indices were negatively associated with the number of years in which mosquito surveillance traps detected at least one WNV-positive pool (Shannon: $\beta=-7.85$, $p=0.046$, $R^{2}=0.51$; Simpson: $\beta=-51.41$, $p=0.034$, $R^{2}=0.56$) (Figs Ba and Bb). No statistically significant relationship was found between bird species richness (Chao2 index) and WNV detection frequency ($\beta=0.04$, $p=0.869$, $R^{2}=0.005$) (Fig Bc).

**Table A. Rarefied diversity indices (Shannon’s, Simpson’s, and Chao2) for eight WNV detection frequency groups based on years with at least one WNV-positive mosquito pool (Exclusion-based Rarefaction).**

| **WNV Detection Frequency (Years)** | **Shannon's Diversity Index** | **Simpson’s Diversity Index** | | **Chao2 Index** |
| --- | --- | --- | --- | --- |
| 1 | 3.073 | | 0.928 | 55.579 |
| 3 | 2.665 | | 0.854 | 61.838 |
| 4 | 3.195 | | 0.918 | 63.061 |
| 6 | 2.823 | | 0.883 | 66.782 |
| 7 | 2.707 | | 0.856 | 61.922 |
| 8 | 2.877 | | 0.894 | 51.216 |
| 9 | 2.406 | | 0.817 | 53.649 |
| 10 | 2.405 | | 0.802 | 68.434 |

**
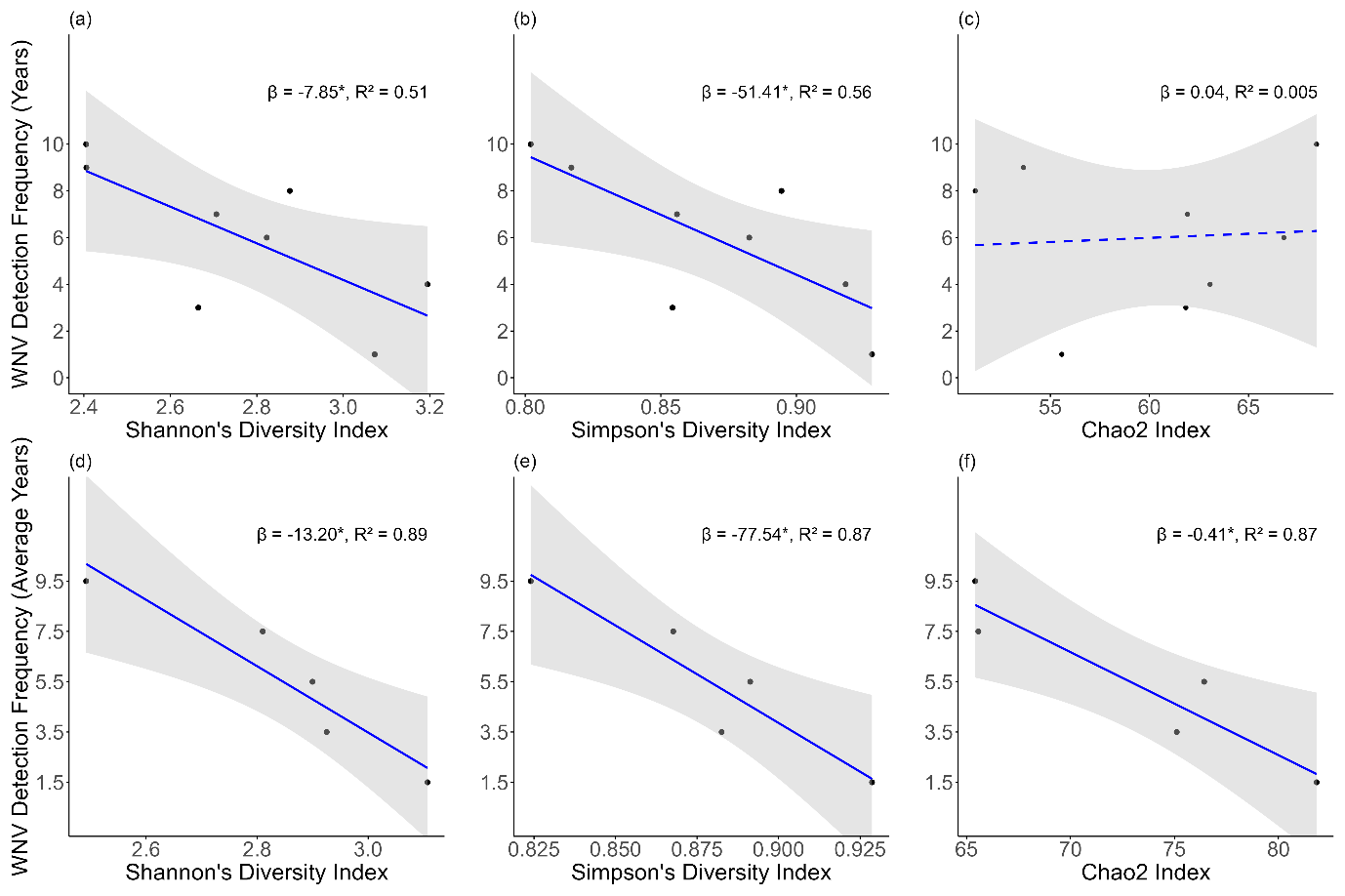
Fig B. Negative associations between rarefied bird community biodiversity indices and WNV detection frequency, measured as the number of years with at least one WNV-positive pool in the Exclusion-based Rarefaction approach (a-c) and the average number of years with at least one WNV-positive pool in the Combination-based Rarefaction approach (d-f), supporting the dilution effect hypothesis.** β and R² represent the coefficient and coefficient of determination from simple linear regression, respectively; * indicating a p-value (p) p≤0.05 and ** indicating p≤0.01. Solid lines were used for p≤0.05, a dashed line denotes a non-significant trend (p>0.05). (a) Rarefied Shannon’s Diversity Index versus WNV detection frequency (Exclusion-based Rarefaction). (b) Rarefied Simpson’s Diversity Index versus WNV detection frequency (Exclusion-based Rarefaction). (c) Rarefied Chao2 Index versus WNV detection frequency (Exclusion-based Rarefaction). (d) Rarefied Shannon’s Diversity Index versus WNV detection frequency (Combination-based Rarefaction). (e) Rarefied Simpson’s Diversity Index versus WNV detection frequency (Combination-based Rarefaction). (f) Rarefied Chao2 Index versus WNV detection frequency (Combination-based Rarefaction).

Table B summarises the Shannon’s, Simpson’s, and Chao2 indexes calculated using the Combination-based Rarefaction approach based on 27 subsamples (Fig Ac). Similar to the Exclusion-based Rarefaction method, the results from simple linear regressions revealed negative associations between Shannon’s and Simpson’s diversity indices and WNV detection frequency (Shannon: $\beta=-13.20$, $p=0.017$, $R^{2}=0.89$; Simpson: $\beta=-77.54$, $p=0.021$, $R^{2}=0.87$) (Figs Bd and Be). A negative relationship was also observed between the Chao2 index and the number of years with at least one WNV-positive pool ($\beta=-0.41$, $p=0.021$, $R^{2}=0.87$) (Fig Bf)*.*

**Table B.** **Rarefied diversity indices (Shannon’s, Simpson’s, and Chao2) for five WNV detection frequency groups based on average number of years with at least one WNV-positive mosquito pool (Combination-based Rarefaction).**

| **WNV Detection Frequency (Average Years)** | **Shannon's Diversity Index** | **Simpson’s Diversity Index** | | **Chao2 Index** |
| --- | --- | --- | --- | --- |
| 1.5 | 3.107 | | 0.929 | 81.831 |
| 3.5 | 2.925 | | 0.883 | 75.110 |
| 5.5 | 2.900 | | 0.891 | 76.426 |
| 7.5 | 2.810 | | 0.868 | 65.570 |
| 9.5 | 2.492 | | 0.824 | 64.424 |
