# Supplementary material for: Association of avian biodiversity and West Nile Virus circulation in Culex mosquitoes in Emilia-Romagna, Italy
Source: PLoS Negl Trop Dis. 2026 Mar 6;20(3):e0014076. doi: 10.1371/journal.pntd.0014076 (PMC12978567; doi:10.1371/journal.pntd.0014076)
Supplement: S3 Text — (DOCX) [file pntd.0014076.s003.docx]

**S3 Text. Assessing spatial autocorrelation in Vector Index**

To evaluate spatial dependence in WNV transmission risk, we calculated the mean Vector Index (VI) for each trap across all weeks of the surveillance season. For each trap location, we identified the six nearest neighbouring traps based on distance between the coordinates and computed Moran's I statistic using the *spdep* R package [1]. Moran's I values range from -1 to +1, with positive values indicating positive spatial autocorrelation when accompanied by significant p-values (<0.05).

We found significant spatial autocorrelation in VI across trap locations (Moran's I = 0.105, p = 0.022), suggesting that WNV transmission risk exhibits spatial clustering and supporting our modelling assumption that VI follows a continuous spatially correlated process.

**Reference**

1. Pebesma, E., Bivand R. Spatial Data Science: With Applications in R (1st ed.). Chapman and Hall/CRC.; 2023.
