# Supplementary material for: Association of avian biodiversity and West Nile Virus circulation in Culex mosquitoes in Emilia-Romagna, Italy
Source: PLoS Negl Trop Dis. 2026 Mar 6;20(3):e0014076. doi: 10.1371/journal.pntd.0014076 (PMC12978567; doi:10.1371/journal.pntd.0014076)
Supplement: S4 Text — (DOCX) [file pntd.0014076.s004.docx]

**S4 Text. Mesh construction and prior specifications of the INLA-SPDE model**

The spatial random field in the model was built on a triangulated mesh encompassing Emilia-Romagna and neighbouring regions. The boundary for the field on the mesh was set to include the coastal border of the region using a Global Administrative Areas (GADM) shapefile [1]. To maintain regularity in the shape and size of the triangles, the mesh was constrained to a minimum triangle angle of 25°. The maximum edge length of the triangles was determined by the spatial distribution of trap locations, with finer resolution near the boundary to minimize potential boundary effects (Fig A).

**
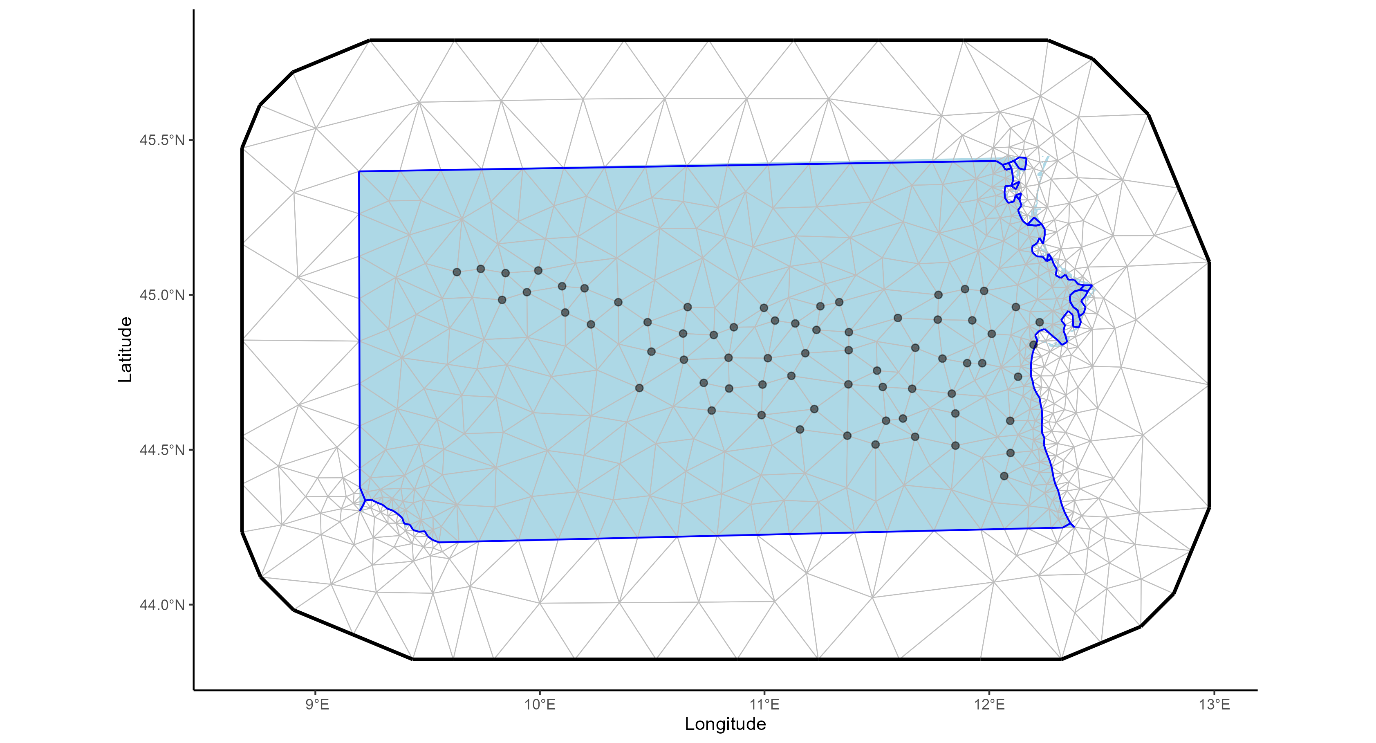
Fig A. Triangulated mesh used to represent the region and build the random field upon.** Black dots represent the 68 mosquito traps active during the 2013-2023 surveillance seasons in Emilia-Romagna, Italy. The blue line and contained shaded region denote the random field boundary.

The spatial random field is a solution to the SPDEs specifying the spatial correlation between any two locations, and is characterised by the Matérn covariance function [2, 3], with a smoothness parameter of 2, and two hyperparameters: the range of the spatial correlation ($\rho$), and the marginal standard deviation ($\sigma$). Priors for these hyperparameters were defined using penalized complexity priors [4, 5], specified to be weakly informative to allow sufficient flexibility. Specifically, the prior for the range was set such that $P\left( \rho<d_{min} \right)=0.5$, indicating a 50% probability that the spatial correlation range is smaller than the minimum distance between any two mosquito traps ($d_{min}$). For the standard deviation, the prior was defined as $P\left( \sigma>d_{min} \right)=0.5,$indicating a 50% probability that $\sigma$ exceeds the minimum inter-trap distance.

**References**

1. Berkely UoC. Database of Global Administrative Boundaries Version 3.6. published 6 May 2018 [cited 23 June 2025]. Database: GADM [Internet]. Available from: <https://gadm.org>
2. G, Auteri L, Pavan V, Tomei F, Tomozeiu R, Marletto V. A daily high-resolution gridded climatic data set for Emilia-Romagna, Italy, during 1961-2010. Int J Climatol. 2016;36(4):1970-86. doi: 10.1002/joc.4473. PubMed PMID: WOS:000372036800030.
3. Bakka H, Rue H, Fuglstad GA, Riebler A, Bolin D, Illian J, et al. Spatial modeling with R-INLA: A review. Wires Comput Stat. 2018;10(6). doi: 10.1002/wics.1443. PubMed PMID: WOS:000447164400003.
4. Simpson D, Rue H, Riebler A, Martins TG, Sorbye SH. Penalising Model Component Complexity: A Principled, Practical Approach to Constructing Priors. Stat Sci. 2017;32(1):1-28. doi: 10.1214/16-Sts576. PubMed PMID: WOS:000399375400001.
5. Fuglstad GA, Simpson D, Lindgren F, Rue H. Constructing Priors that Penalize the Complexity of Gaussian Random Fields. J Am Stat Assoc. 2019;114(525):445-52. doi: 10.1080/01621459.2017.1415907. PubMed PMID: WOS:000471325500039.
