# Supplementary material for: Association of avian biodiversity and West Nile Virus circulation in Culex mosquitoes in Emilia-Romagna, Italy
Source: PLoS Negl Trop Dis. 2026 Mar 6;20(3):e0014076. doi: 10.1371/journal.pntd.0014076 (PMC12978567; doi:10.1371/journal.pntd.0014076)
Supplement: S1 Table — β refers to regression coefficients, 95% CI refers to 95% confidence intervals. (DOCX) [file pntd.0014076.s005.docx]

**S1 Table.** **Rarefied biodiversity indices (Shannon’s, Simpson’s, and Chao2) by eight WNV detection frequency groups and regression analysis results (Exclusion-based Rarefaction).** $\beta$ refers to regression coefficients, 95% CI refers to 95% confidence intervals.

| **WNV Detection Frequency (Years)** | **Shannon's Diversity Index** | **Simpson’s Diversity Index** | | **Chao2 Index** | |
| --- | --- | --- | --- | --- | --- |
| 1 | 3.213 | | 0.931 | | 73.931 |
| 3 | 2.956 | | 0.902 | | 68.111 |
| 4 | 3.240 | | 0.921 | | 62.634 |
| 6 | 2.884 | | 0.890 | | 78.475 |
| 7 | 2.745 | | 0.860 | | 69.128 |
| 8 | 2.918 | | 0.898 | | 54.264 |
| 9 | 2.436 | | 0.821 | | 59.991 |
| 10 | 2.447 | | 0.806 | | 69.962 |
| $\boldsymbol{\beta}$ **(95% CI)** | -8.89 [-14.01, -3.76] | | -59.04 [-93.21, -24.87] | | -0.15 [-0.52, 0.23] |
| **p-value** | 0.005 | | 0.005 | | 0.376 |
| **R²** | 0.75 | | 0.75 | | 0.13 |
