# Supplementary material for: Association of avian biodiversity and West Nile Virus circulation in Culex mosquitoes in Emilia-Romagna, Italy
Source: PLoS Negl Trop Dis. 2026 Mar 6;20(3):e0014076. doi: 10.1371/journal.pntd.0014076 (PMC12978567; doi:10.1371/journal.pntd.0014076)
Supplement: S2 Table — β refers to regression coefficients, 95% CI refers to 95% confidence intervals. (DOCX) [file pntd.0014076.s006.docx]

**S2 Table. Rarefied biodiversity indices (Shannon’s, Simpson’s, and Chao2) by five WNV detection frequency groups and regression analysis results (Combination-based Rarefaction).** $\beta$ refers to regression coefficients, 95% CI refers to 95% confidence intervals.

| **WNV Detection Frequency (Average Years)** | **Shannon's Diversity Index** | **Simpson’s Diversity Index** | **Chao2 Index** |
| --- | --- | --- | --- |
| 1.5 | 3.289 | 0.930 | 102.442 |
| 3.5 | 3.123 | 0.913 | 87.278 |
| 5.5 | 2.996 | 0.903 | 94.080 |
| 7.5 | 2.874 | 0.872 | 82.513 |
| 9.5 | 2.545 | 0.828 | 76.347 |
| $\boldsymbol{\beta}$ **(95% CI)** | -11.01 [-15.35, -6.67] | -76.15 [-113.80, -38.50] | -0.28 [-0.54, -0.01] |
| **p-value** | 0.004 | 0.008 | 0.044 |
| **R²** | 0.96 | 0.93 | 0.79 |
