# Supplementary material for: Association of avian biodiversity and West Nile Virus circulation in Culex mosquitoes in Emilia-Romagna, Italy
Source: PLoS Negl Trop Dis. 2026 Mar 6;20(3):e0014076. doi: 10.1371/journal.pntd.0014076 (PMC12978567; doi:10.1371/journal.pntd.0014076)
Supplement: S3 Table — The entry ‘21’ in column ‘Version’ indicates the 2021 version of eBird (based on data from 2007–2021) and ‘22’ indicates the 2022 version (based on data from 2008–2022). The species classified as fully migratory are highlighted in bold. (DOCX) [file pntd.0014076.s007.docx]

**S3 Table. Bird species in Emilia-Romagna, Italy with available weekly abundance estimates from May to September to download from the eBird website.** The entry '21' in column ‘Version’ indicates the 2021 version of eBird (based on data from 2007–2021) and '22' indicates the 2022 version (based on data from 2008–2022). The species classified as fully migratory are highlighted in bold.

| **English name** | **Order** | **eBird reference** | **Version** |
| --- | --- | --- | --- |
| Alpine Swift | Apodiformes | alpswi1 | 21 |
| **Arctic Loon** | Gaviiformes | arcloo | 21 |
| Barn Swallow | Passeriformes | barswa | 22 |
| **Bar-tailed Godwit** | Charadriiformes | batgod | 22 |
| Black Kite | Accipitriformes | blakit1 | 22 |
| Black Redstart | Passeriformes | blared1 | 22 |
| **Black Stork** | Ciconiiformes | blasto1 | 21 |
| Black Tern | Charadriiformes | blkter | 22 |
| **Black-bellied Plover** | Charadriiformes | bkbplo | 22 |
| Black-crowned Night-heron | Pelecaniformes | bcnher | 22 |
| Black-headed Gull | Charadriiformes | bkhgul | 21 |
| **Black-necked Grebe** | Podicipediformes | eargre | 22 |
| Black-winged Stilt | Charadriiformes | bkwsti | 21 |
| **Bluethroat** | Passeriformes | blueth | 22 |
| **Booted Eagle** | Accipitriformes | booeag1 | 22 |
| **Brambling** | Passeriformes | brambl | 21 |
| Carrion Crow | Passeriformes | carcro1 | 21 |
| Caspian Tern | Charadriiformes | caster1 | 22 |
| Cattle Egret | Pelecaniformes | categr | 22 |
| **Collared Flycatcher** | Passeriformes | colfly1 | 21 |
| Collared Sand Martin | Passeriformes | banswa | 22 |
| Common Chaffinch | Passeriformes | comcha | 22 |
| Common Chiffchaff | Passeriformes | comchi1 | 22 |
| Common Coot | Gruiformes | eurcoo | 21 |
| Common Cuckoo | Cuculiformes | comcuc | 21 |
| Common Firecrest | Passeriformes | firecr1 | 21 |
| **Common Grasshopper Warbler** | Passeriformes | cogwar1 | 21 |
| **Common Greenshank** | Charadriiformes | comgre | 21 |
| Common Gull-billed Tern | Charadriiformes | gubter1 | 22 |
| Common Hoopoe | Bucerotiformes | hoopoe | 21 |
| Common Kestrel | Falconiformes | eurkes | 22 |
| Common Kingfisher | Coraciiformes | comkin1 | 22 |
| Common Moorhen | Gruiformes | commoo3 | 21 |
| Common Nightingale | Passeriformes | comnig1 | 22 |
| Common Pheasant | Galliformes | rinphe1 | 22 |
| Common Redstart | Passeriformes | comred2 | 21 |
| Common Reed Warbler | Passeriformes | eurwar1 | 21 |
| **Common Ringed Plover** | Charadriiformes | corplo | 21 |
| Common Sandpiper | Charadriiformes | comsan | 22 |
| Common Shelduck | Anseriformes | comshe | 21 |
| **Common Snipe** | Charadriiformes | comsni | 21 |
| Common Starling | Passeriformes | eursta | 22 |
| Common Stonechat | Passeriformes | stonec4 | 21 |
| Common Swift | Apodiformes | comswi | 21 |
| Common Tern | Charadriiformes | comter | 22 |
| Common Whitethroat | Passeriformes | grewhi1 | 22 |
| Common Woodpigeon | Columbiformes | cowpig1 | 22 |
| **Dunlin** | Charadriiformes | dunlin | 22 |
| **Dunnock** | Passeriformes | dunnoc1 | 21 |
| Eurasian Blackbird | Passeriformes | eurbla | 22 |
| Eurasian Blackcap | Passeriformes | blackc1 | 21 |
| Eurasian Blue Tit | Passeriformes | blutit | 22 |
| Eurasian Bullfinch | Passeriformes | eurbul | 21 |
| Eurasian Buzzard | Accipitriformes | combuz1 | 21 |
| Eurasian Collared-Dove | Columbiformes | eucdov | 22 |
| Eurasian Crag-Martin | Passeriformes | eurcrm1 | 22 |
| Eurasian Golden Oriole | Passeriformes | eugori2 | 22 |
| Eurasian Jay | Passeriformes | eurjay1 | 22 |
| Eurasian Linnet | Passeriformes | eurlin1 | 21 |
| Eurasian Magpie | Passeriformes | eurmag1 | 22 |
| Eurasian Siskin | Passeriformes | eursis | 21 |
| Eurasian Skylark | Passeriformes | skylar | 21 |
| Eurasian Sparrowhawk | Accipitriformes | eurspa1 | 21 |
| Eurasian Tree Sparrow | Passeriformes | eutspa | 22 |
| Eurasian Wigeon | Anseriformes | eurwig | 21 |
| Eurasian Wryneck | Piciformes | eurwry | 21 |
| European Bee-eater | Coraciiformes | eubeat1 | 22 |
| European Goldfinch | Passeriformes | eurgol | 22 |
| European Greenfinch | Passeriformes | eurgre1 | 21 |
| European Honey-buzzard | Accipitriformes | euhbuz1 | 21 |
| **European Pied Flycatcher** | Passeriformes | eupfly1 | 21 |
| European Robin | Passeriformes | eurrob1 | 22 |
| European Roller | Coraciiformes | eurrol1 | 21 |
| European Serin | Passeriformes | eurser1 | 22 |
| European Turtle-Dove | Columbiformes | eutdov | 21 |
| Feral Pigeon | Columbiformes | rocpig | 22 |
| Gadwall | Anseriformes | gadwal | 22 |
| Garganey | Anseriformes | gargan | 21 |
| Glossy Ibis | Pelecaniformes | gloibi | 22 |
| **Golden Eagle** | Accipitriformes | goleag | 22 |
| Great Reed Warbler | Passeriformes | grrwar1 | 21 |
| Great Tit | Passeriformes | gretit1 | 22 |
| Great White Egret | Pelecaniformes | greegr | 22 |
| **Greater White-fronted Goose** | Anseriformes | gwfgoo | 22 |
| **Green Sandpiper** | Charadriiformes | grnsan | 21 |
| Green-winged Teal | Anseriformes | gnwtea | 22 |
| Grey Heron | Pelecaniformes | graher1 | 21 |
| Grey Partridge | Galliformes | grypar | 22 |
| Grey Wagtail | Passeriformes | grywag | 22 |
| Greylag Goose | Anseriformes | gragoo | 21 |
| **Hen Harrier** | Accipitriformes | norhar1 | 21 |
| Hooded Crow | Passeriformes | hoocro1 | 21 |
| **Icterine Warbler** | Passeriformes | ictwar1 | 21 |
| Lesser Grey Shrike | Passeriformes | legshr2 | 21 |
| Lesser Redpoll | Passeriformes | lesred1 | 21 |
| **Lesser Whitethroat** | Passeriformes | leswhi4 | 22 |
| **Little Gull** | Charadriiformes | litgul | 21 |
| Little Owl | Strigiformes | litowl1 | 22 |
| Little Ringed Plover | Charadriiformes | lirplo | 22 |
| **Little Stint** | Charadriiformes | litsti | 21 |
| Little Tern | Charadriiformes | litter1 | 21 |
| Mallard | Anseriformes | mallar3 | 22 |
| Marsh Tit | Passeriformes | martit2 | 22 |
| Melodious Warbler | Passeriformes | melwar1 | 21 |
| **Merlin** | Falconiformes | merlin | 22 |
| Mistle Thrush | Passeriformes | misthr1 | 21 |
| Montagu's Harrier | Accipitriformes | monhar1 | 21 |
| Mute Swan | Anseriformes | mutswa | 22 |
| Northern House Martin | Passeriformes | cohmar1 | 22 |
| Northern Lapwing | Charadriiformes | norlap | 22 |
| **Northern Pintail** | Anseriformes | norpin | 22 |
| Northern Wren | Passeriformes | winwre4 | 21 |
| Ortolan Bunting | Passeriformes | ortbun1 | 21 |
| **Osprey** | Accipitriformes | osprey | 22 |
| Peregrine Falcon | Falconiformes | perfal | 22 |
| Purple Heron | Pelecaniformes | purher1 | 21 |
| **Red Kite** | Accipitriformes | redkit1 | 22 |
| **Red Knot** | Charadriiformes | redkno | 22 |
| Red-backed Shrike | Passeriformes | rebshr1 | 22 |
| **Red-breasted Merganser** | Anseriformes | rebmer | 22 |
| Red-legged Partridge | Galliformes | relpar1 | 22 |
| **Red-necked Grebe** | Podicipediformes | rengre | 22 |
| **Red-throated Loon** | Gaviiformes | retloo | 22 |
| **Ruff** | Charadriiformes | ruff | 21 |
| Sandwich Tern | Charadriiformes | santer1 | 22 |
| Sardinian Warbler | Passeriformes | sarwar1 | 22 |
| **Sedge Warbler** | Passeriformes | sedwar1 | 21 |
| **Short-toed Snake-eagle** | Accipitriformes | shteag1 | 21 |
| Short-toed Treecreeper | Passeriformes | shttre1 | 22 |
| Spotted Flycatcher | Passeriformes | spofly1 | 21 |
| **Spotted Redshank** | Charadriiformes | spored | 21 |
| **Stock Dove** | Columbiformes | stodov1 | 21 |
| **Temminck's Stint** | Charadriiformes | temsti | 21 |
| **Tree Pipit** | Passeriformes | trepip | 21 |
| Tufted Duck | Anseriformes | tufduc | 21 |
| **Velvet Scoter** | Anseriformes | whwsco3 | 21 |
| Water Rail | Gruiformes | watrai1 | 21 |
| Western Bonelli's Warbler | Passeriformes | webwar1 | 21 |
| Western Marsh-harrier | Accipitriformes | wemhar1 | 21 |
| Western Yellow Wagtail | Passeriformes | eaywag1 | 21 |
| **Whimbrel** | Charadriiformes | whimbr | 22 |
| White Wagtail | Passeriformes | whiwag | 21 |
| White-winged Tern | Charadriiformes | whwter | 21 |
| **Willow Warbler** | Passeriformes | wlwwar | 22 |
| **Wood Sandpiper** | Charadriiformes | woosan | 21 |
| **Wood Warbler** | Passeriformes | woowar | 21 |
| Woodlark | Passeriformes | woolar1 | 21 |
| Yellowhammer | Passeriformes | yellow2 | 21 |
