# Supplementary material for: Association of avian biodiversity and West Nile Virus circulation in Culex mosquitoes in Emilia-Romagna, Italy
Source: PLoS Negl Trop Dis. 2026 Mar 6;20(3):e0014076. doi: 10.1371/journal.pntd.0014076 (PMC12978567; doi:10.1371/journal.pntd.0014076)
Supplement: S4 Table — (DOCX) [file pntd.0014076.s008.docx]

**S4 Table. Summary of fixed effects from Bayesian spatiotemporal regression examining the association between total bird species richness and Vector Index (VI) representing West Nile Virus (WNV) transmission risk in *Culex* mosquitoes:** posterior means and 95% credible intervals (CrIs) for predictor variables.

| **Variables** | **Mean** | **2.5% CrI** | **97.5% CrI** |
| --- | --- | --- | --- |
| Total bird species richness (lag 3) | 0.000 | -0.001 | 0.001 |
| CLC2 (Agricultural area) | 0.802 | 0.091 | 1.521 |
| Weekly Average Temperature (lag 3) | 0.147 | 0.145 | 0.149 |
| Cumulative precipitation (lag 0-2) | -0.009 | -0.010 | -0.009 |
| Weekly Average Solar Radiation (lag 1) | 0.027 | 0.025 | 0.029 |
