# Supplementary material for: Association of avian biodiversity and West Nile Virus circulation in Culex mosquitoes in Emilia-Romagna, Italy
Source: PLoS Negl Trop Dis. 2026 Mar 6;20(3):e0014076. doi: 10.1371/journal.pntd.0014076 (PMC12978567; doi:10.1371/journal.pntd.0014076)
Supplement: S5 Table — (DOCX) [file pntd.0014076.s009.docx]

**S5 Table. Summary of fixed effects from Bayesian spatiotemporal regression examining the association between fully migratory bird species richness and Vector Index (VI) representing West Nile Virus (WNV) transmission risk in *Culex* mosquitoes:** posterior means and 95% credible intervals (CrIs) for predictor variables.

| **Variables** | **Mean** | **2.5% CrI** | **97.5% CrI** |
| --- | --- | --- | --- |
| Fully migratory bird species richness (lag 3) | -0.034 | -0.035 | -0.033 |
| CLC2 (Agricultural area) | 0.941 | 0.217 | 1.671 |
| Weekly Average Temperature (lag 3) | 0.150 | 0.148 | 0.152 |
| Cumulative precipitation (lag 0-2) | -0.008 | -0.008 | -0.007 |
