# Supplementary material for: Association of avian biodiversity and West Nile Virus circulation in Culex mosquitoes in Emilia-Romagna, Italy
Source: PLoS Negl Trop Dis. 2026 Mar 6;20(3):e0014076. doi: 10.1371/journal.pntd.0014076 (PMC12978567; doi:10.1371/journal.pntd.0014076)
Supplement: Attachment — Submitted filename: Reviewers comments.doc [file pntd.0014076.s010.doc]

### The manuscript entitled “Association of avian biodiversity and West Nile virus circulation in mosquitoes in Emilia-Romagna, Italy” is a very interesting study as it describes the zooprophylactic effect of avian biodiversity on West Nile virus circulation in mosquitoes in endemic foci located in Northern Italy. Several points mainly must be addressed before considering this manuscript for publication. Thus, I recommend that the authors should address the following comments before considering this manuscript for publication.

**Introduction**

The DEH has been demonstrated in multiple vector-borne zoonotic diseases systems such … Please include the following article:

Chelbi, I. B. Kaabi, M. Derbali, S. Ben Hadj Ahmed, K. Dellagi, and E. Zhioua. 2008. Zooprophylaxis: Impact of breeding rabbits in man-made underground holes around houses on reducing the indoor density of *Phlebotomus papatasi*, principal vector of *Leishmania major*, etiologic agent of zoonotic cutaneous leishmaniasis in the Old World. Vector-Borne and Zoonotic Diseases, 8: 741-747.

**Materials and methods**

I have one comment regarding the calculation of "Biodiversity indicators": Authors used point-count survey with unlimited distances to infer and compare the occurrence and abundance of bird species. However, such a method provides bird detection data that could not be used in interspecific comparisons because of possible differences in detectability among species. Highly conspicuous species may be more detectable than expected by their abundance, and vice versa. By using the chao estimator, this detectability issue has nicely been taken into account in the estimation of species richness. However, this issue was completely ignored when calculating the Shannon and Simpson diversity indices (both are based on relative abundance data), possibly leading to biased estimates. Therefore, I'm wondering if there is a way to convert detections into abundance data? Are there species-specific correction coefficients that may be used to convert the detection data into number of individuals?

**Discussion**

The authors should discuss how bird migration from North Africa which is endemic for WNV Northward to Italy impact WNV circulation.

From 2013 to 2023, major outbreak of WNND occurred during 2018 in this foci. The author should discuss the relationship between entomological surveillance data and ornithological data sources during 2018.

**Conclusion**

There are conflicting findings regarding the impact of host biodiversity leading either to zooprophylactic or zoopotentiation. Taking into account the size of this study by investigating the effect of avian biodiversity on WNV circulation in *Culex* mosquito populations by analysing 11 years of entomological surveillance data and two ornithological data sources, the findings provide strong evidence of the DEH of WNV in this endemic focus.
