# Supplementary material for: Association of avian biodiversity and West Nile Virus circulation in Culex mosquitoes in Emilia-Romagna, Italy
Source: PLoS Negl Trop Dis. 2026 Mar 6;20(3):e0014076. doi: 10.1371/journal.pntd.0014076 (PMC12978567; doi:10.1371/journal.pntd.0014076)
Supplement: Attachment — Submitted filename: Response to Reviewers.docx [file pntd.0014076.s012.docx]

**Association of avian biodiversity and West Nile Virus circulation in Culex mosquitoes in Emilia-Romagna, Italy**

**Point-by-point response letter**

We would like to thank the Editors and Reviewers for their valuable feedback and constructive comments. Below, the editor and reviewer comments are presented in black and numbered sequentially. Our responses are provided in blue, and quoted text from the manuscript appears in italics.

**Major methodological change:**

In response to Reviewer 6’s comments on the importance of accounting for local mosquito abundance when relating avian biodiversity to WNV transmission, we implemented a major methodological change in the eBird data analysis. Specifically, we changed the outcome indicator in the spatiotemporal analysis of the eBird data from the proportion of mosquito pools testing positive to the Vector Index (VI), which combines the estimated infection rate in pooled samples with local mosquito abundance per trap-night and therefore reflects the relative density of infected mosquitoes in the population, providing a more informative indicator of WNV transmission risk. We re-ran the spatiotemporal models using VI as the outcome indicator and updated the Methods, Results, Discussion, and the relevant tables and figures accordingly.

**Journal Requirements:**

If the reviewer comments include a recommendation to cite specific previously published works, please review and evaluate these publications to determine whether they are relevant and should be cited. There is no requirement to cite these works unless the editor has indicated otherwise.

Thank you for the suggestion. The reviewer recommended only one publication. We have reviewed it, agreed that it enriches our manuscript, and have included it in the revised version.

1) Some material included in your submission may be copyrighted. According to PLOSu2019s copyright policy, authors who use figures or other material (e.g., graphics, clipart, maps) from another author or copyright holder must demonstrate or obtain permission to publish this material under the Creative Commons Attribution 4.0 International (CC BY 4.0) License used by PLOS journals. Please closely review the details of PLOSu2019s copyright requirements here: PLOS Licenses and Copyright. If you need to request permissions from a copyright holder, you may use PLOS's Copyright Content Permission form.
Please respond directly to this email and provide any known details concerning your material's license terms and permissions required for reuse, even if you have not yet obtained copyright permissions or are unsure of your material's copyright compatibility. Once you have responded and addressed all other outstanding technical requirements, you may resubmit your manuscript within Editorial Manager.

Thank you very much for raising this potential copyright issue. We responded to the email to address this and summarise the main information here for your reference:

We checked that the basemap for the original Figure 1 was sourced from OpenStreetMap, which is licensed under CC BY-SA 2.0. While this is open data, we recognise that the CC BY-SA 2.0 license is not fully compatible with PLOS's CC BY 4.0 licensing requirements.

To ensure full compliance with PLOS copyright policy, we have re-created Figure 1 using Natural Earth data (<https://www.naturalearthdata.com/>), as recommended in your guidelines. The new figure was generated using the R packages *rnaturalearth* and *rnaturalearthdata*. We have also updated the figure caption in the manuscript to include: "Base map from Natural Earth (public domain, naturalearthdata.com)." The updated version of Figure 1 is shown below:

*Fig 1. Map of WNV mosquito surveillance traps active from 2013 to 2023 (points, color-coded from purple to yellow by WNV detection frequency, classified by the number of years with at least one mosquito pool testing WNV positive) in Emilia-Romagna (highlighted yellow on the map of Italy), with corresponding buffer areas (grey circles) and bird observational stations (red squares). Some traps do not have associated buffer areas because no matched bird observation data were available. Base map from Natural Earth (public domain, naturalearthdata.com).*

2) Please amend your detailed Financial Disclosure statement. This is published with the article. It must therefore be completed in full sentences and contain the exact wording you wish to be published.

1) State the initials, alongside each funding source, of each author to receive each grant. For example: "This work was supported by the National Institutes of Health (####### to AM; ###### to CJ) and the National Science Foundation (###### to AM)."

2) State what role the funders took in the study. If the funders had no role in your study, please state: "The funders had no role in study design, data collection and analysis, decision to publish, or preparation of the manuscript."

3) If any authors received a salary from any of your funders, please state which authors and which funders.

If you did not receive any funding for this study, please simply state: u201cThe authors received no specific funding for this work.

We have included the following statement (pages 17-18, lines 587-600), according to the instructions that we have received:

*“**The WNV data were collected as part of the activities conducted by the Emilia-Romagna Regional Plan for Surveillance and Control of Arboviruses. The Bird observation data were collected by Lipu (Lega Italiana Protezione Uccelli and Italian BirdLife partner) as part of the Farmland Bird Index project, funded by the Ministry of Agriculture, Food Sovereignty and Forestry through the NRN 2014/2022 programme, EAFRD - European Agricultural Fund for Rural Development. YW acknowledges funding from the NERC Grantham Institute SSCP DTP (grant number NE/S007415/1). YW, VC, SJ and ID acknowledge funding from the MRC Centre for Global Infectious Disease Analysis (reference MR/X020258/1), funded by the UK Medical Research Council (MRC). This UK funded award is carried out in the frame of the Global Health EDCTP3 Joint Undertaking. ID and SJ acknowledge research funding from Welcome Trust (grant 213494/Z/18/Z and 228185/Z/23/Z, 226727/Z/22/Z). The funders had no role in study design, data collection and analysis, decision to publish, or preparation of the manuscript.”*

**Reviewers' Comments:**

**Reviewer #1:**

**Summary and General Comments**

The manuscript entitled: Association of avian biodiversity and West Nile Virus circulation in Culex mosquitoes in Emilia-Romagna, Italy is a well written and structured, comprehensive and scientifically sound paper. It presents with a clear rationale linking biodiversity and WNV transmission within the dilution effect hypothesis framework. The manuscript benefits from the integration of entomological data from consecutive years along with multiple avian datasets and the use of innovative Bayesian models and rarefaction to control sampling bias, achieving balance of ecological and epidemiological approaches.

We thank the Reviewer for the positive feedback.

General comments for improvement
Language: Some sentences are long and complicated, difficult to follow; shortening them or splitting in 2-3 sentences would improve readability.
Keep focused on the key messages: Τhe authors, in certain points stress too many the methodological details and deviate from the aim of the study especially in the Discussion section, where interpretation of the results is what the reader expects.

Thank you for the constructive feedback. We have revised the manuscript to improve readability by shortening long sentences and improving the flow of the text, particularly in the Discussion section, where we clarified the key messages and reduced unnecessary methodological detail.

Consistency in use of terminology: Always use “avian biodiversity, “instead of “bird diversity,” or “species richness,” DEH after its first use and so on.

Thank you for pointing this out. We have modified our manuscript to use this term consistently when discussing our overall findings and key take-home messages. However, our two analyses employ different biodiversity indicators that capture distinct dimensions of biodiversity: the Farmland Bird Index analysis uses Shannon and Simpson diversity indices (which incorporate relative abundance), whereas the eBird analysis is based solely on species richness metrics (and does not incorporate abundance data). Given these methodological differences, we preferred to retain this accuracy when describing individual associations, but we used "avian biodiversity" for our broader conclusions as suggested.

Figures and Tables: The Figure references, legends and captions could be slightly simplified to help the readers perceive the key points and results.

Thank you for the suggestion, we have done our best to simplify the figure references and captions as suggested.

Detailed comments
The Abstract section could be shortened by rephrasing, e.g. Instead of “using 11 consecutive years…” say “based on 11 years…”. “Non-passerine species richness was negatively associated with WNV circulation (supporting the DEH), while passerine richness showed a positive association after adjustment.” The last sentence: These findings have important implications…, could end somehow with a stronger effect:…informing biodiversity conservation and integrated public health surveillance activities in Europe.”
The Author Summary section could also benefit with merging or rephrasing, as it repeats content from the abstract and with the addition in the end of a key message.
The Introduction section is comprehensive but rather dense. The authors should consider reducing it by 400-500 words. For example, the first two paragraphs on WNV ecology and vector–host dynamics could be merged to make the manuscript flow for the reader. The 3rd paragraph on biodiversity definitions could be shortened by summarizing the three metric characteristics in one sentence.

Thank you for the feedback on improving readability. We have incorporated the suggestions by rephrasing parts of the Abstract and Author Summary to make them more concise and clearer.

The Methods section is detailed and organized, however the authors should consider transferring the long equations and the detailed rarefaction explanations to Supplementary section.

Thank you for these helpful suggestions. We have moved the equations for the biodiversity indicators to the Supplementary Information (S1 Text). However, on balance, we think that it will be clearer for the readers if we keep the rarefaction details in the main text so that there is an easy to find reference for the two different approaches (“combination-based rarefaction” vs “exclusion-based rarefaction”).

Also, please explain and add relevant references regarding the rationale for including a 3 km buffer and cite the mosquito flight range radius somewhere before in the text.

We have also added a clearer justification for the choice of the 3-km buffer as suggested. The Methods section now reads as follows (page 5, lines 180-183):

*“Given that Culex mosquitoes have been documented to fly up to 2 miles (~ 3.2 km) [43], bird observation records were extracted from sampling units located within a 3-km buffer of the selected mosquito traps to ensure that the bird observations represent the avian communities most likely to be encountered by local mosquitoes (Fig 1).”*

Furthermore, when describing INLA–SPDE, a few sentences explaining why, how, this model was chosen and adapted would help the non specialist readers to further understand.

Thank you for highlighting the importance of explaining the INLA-SPDE framework for non-specialist readers. We expanded the Methods section to clarify why this approach was selected and how it was applied in our analysis. We also now cite a recently published study applying the same analytical framework to mosquito surveillance data in Emilia-Romagna, which provides additional methodological context. The revised text reads (page 9, lines 292-305):

*“A Bayesian spatiotemporal model [58] was adapted to examine the relationships between WNV transmission risk and bird species richness, adjusting for environmental covariates in a seasonal context using the Integrated Nested Laplace Approximation - Stochastic Partial Differential Equation (INLA-SPDE) approach implemented in R-INLA (www.r-inla.org) [59, 60]. This approach was chosen because it efficiently models spatially structured ecological processes from irregularly spaced sampling locations, as in the mosquito surveillance network analysed.*

*WNV transmission risk, as measured by VI, was assumed to be a continuous, spatially correlated process across the region. This assumption was validated through spatial autocorrelation analysis, which revealed significant clustering of VI (see S3 Text). The spatial process was represented using a discretized Gaussian Markov random field (GMRF) on a triangulated mesh [61, 62]. The model combines the spatial field (a random effect) with the avian, meteorological, and land use variables (fixed effect covariates) to reconstruct the observed patterns in mosquito WNV circulation.”*

The Results section includes a logical progression to the study’s findings. The authors should ensure that the Figure legends summarize findings, not only a description of content.
E.g. in Figure 3 it should be stated that Higher Shannon and Simpson diversity indices were associated with fewer WNV-positive years, supporting the dilution effect. In addition, the authors should consider presenting in Tables the main regression outputs such as p-values to aid the readers in interpreting.

Thank you for these helpful suggestions. We have updated the legend of Figure 3 to summarise the key findings supporting the dilution effect rather than only describing the content. We have also included the main regression outputs including coefficients, 95% confidence intervals, and p-values in Tables S1 and S2 for the exclusion-based and combination-based rarefaction analyses, respectively.

Furthermore, in eBird analysis, the authors should explain briefly why passerine vs non-passerine distinction is important, before presenting the results.

We have added a brief explanation in the first paragraph of the eBird results to clarify why we distinguish between passerine and non-passerine species, as suggested (page 12, lines 407-412):

*“Bird species were stratified into passerines or non-passerines given that passerines are generally considered competent hosts that can amplify WNV transmission, whereas non-passerines are considered less competent and may dilute its transmission. Throughout the season, non-passerine species consistently outnumber passerine species around the mosquito surveillance traps. Among the 45 bird species classified as fully migratory (see S3 Table), only 12 were passerines.”*

The Discussion section is informative, including prior literature and methodological explanations. However, it is very thorough and it could be shortened without losing robustness and meaning. The paragraphs beginning with “Additionally, our study provides statistical evidence…” and “In terms of environmental factors…” should be closer together in the text, to group mechanistic interpretations. In Discussion the authors should emphasize on novelty, stress that it is the first empirical DEH evidence for WNV in Europe, integration of two avian databases and sources, and 11-year entomological surveillance study. The last three paragraphs starting from To date…could be rewritten aiming to a more concise implications and future work section, including insights for policy and biodiversity conservation, for WNV surveillance and modelling and suggestions for future research.

Thank you for the valuable and constructive suggestions, we have extensively revised the Discussion as suggested and also emphasized the novelty and importance of our study (page 17, lines 562-567):

*“The results presented in this study provide the first empirical evidence supporting the DEH for WNV transmission in a European setting. This analysis represents one of the most comprehensive studies examining avian biodiversity effects on vector-borne zoonosis transmission from 11 years of systematic entomological surveillance and multiple ornithological data sources, which has important implications for public health practice and biodiversity conservation.”*

Finally, we revised the last three paragraphs to improve the structure of the Discussion, so they more clearly address the interpretation of contrasting results, implications for public health practice and biodiversity conservation, and directions for future research, respectively.

Minor comments
Abbreviations: Define “DEH” at the first mention in Abstract and again in Introduction for clarity and then use only DEH.
Grammar/ English style recommendations:
Replace “comprehensively covers” with “covers comprehensively”
“analysis were conducted” with “analyses were conducted”
Avoid repetitions of “higher/lower levels of WNV circulation”; use instead “reduced/increased WNV activity.”

Thank you for the detailed suggestions, we have adjusted the phrasing as recommended, and we have improved the grammar and style. We have defined “DEH” at the first mention in the Abstract, Author Summary, and Introduction, and then used the abbreviation thereafter.

**Reviewer #3:**

**Methods**

The authors drew upon a national avian monitoring system, upon which they could extract data relevant to routine mosquito/pathogen monitoring stations; as well as the eBird database, which has advantages and disadvantages, as mentioned by the authors. The mosquito sampling represents one of the best organized regional mosquito surveillance networks in Europe. They account for sampling biases in questing vs. gravid traps by a clever derivation of their data (“intensity of circulation” = number of years with at least one positive pool).

**Results**

The results are clearly presented. See below for recommendations to improve.

Thank you for the positive feedback. We have addressed all recommendations below.

**Discussion**

For me the discussion required at least three read-throughs to see that the authors addressed all of the concerns that immediately came to my mind upon reading/understanding the data. My suggestions here and below are to clearly provide justification for the analyses, and make sure the connections between the two main analyses are clearly and simply stated. Maybe it is not possible, in which case it is simply an issue of writing style.

We thank the Reviewer for the constructive feedback. We have revised the discussion to improve coherence and clarity, focusing on smoother transitions between the analyses. We hope the revised version is more readable.

**Editorial and Data Presentation Modifications?**

1. Figure 3 and accompanying text (sorry - there are no line or page numbers, so it is difficult to specify). please include a measure of goodness-of-fit for these regression results. I recommend a simple R-squared coefficient of determination, also on the figure panels, themselves, under the parameter estimate.

Thank you for the suggestion regarding the R-squared coefficients and apologies for the oversight on the page and line numbers – these have been added in the revised version to facilitate review. We have also added R-squared coefficients of determination to assess the goodness-of-fit for the regression results. The R-squared values are now included both in the main text and directly on Figure 3 panels for ease of reference. We have also updated the corresponding figure in the supplementary materials (S1 Text) accordingly.

2. Figure 4. I don’t see the “clear spatiotemporal heterogeneity” mentioned in the text. Perhaps the authors could make this clearer (e.g., is there really a difference in richness between a migration-month and non migration-month?)

Thank you for this comment. We have revised the description to more explicitly outline both dimensions of spatiotemporal heterogeneity: spatially, species richness varies widely across the 68 trap locations each week, as indicated by the error bars representing variation among trap locations; temporally, species richness shows distinct seasonal patterns corresponding to migration periods, with a decline in May as pre-breeding migration ends, relatively stable richness during the June–July breeding season, and a clear increase in August–September during postnuptial migration. The revised text now reads (page 12, lines 398-407):

*“The weekly species richness from May to September at 68 mosquito surveillance traps, active throughout all years, derived from the eBird weekly abundance estimates of 148 bird species in the Emilia-Romagna region, exhibits clear spatiotemporal heterogeneity (Fig 4). Spatially, species richness varies widely across trap locations each week. Temporally, species richness is higher in correspondence with the migration periods, when several non-breeding species stopover in the study area for refuelling. At the beginning of the study period in May, the pre-breeding migration is at its final stage, which translates into a slight decline of species richness. From June to July, richness remains relatively stable, coinciding with the end of the breeding season, and then increases again between August and September, due to postnuptial migration.”*

3. Discussion – I recommend referring to the NY99 “strain” more generally as the “North American WNV lineage 1”. However, it raises and important issue – if the authors want to argue that there may be differences between WNV-1 and WNV-2 within the same geographic region (as they do somewhere in the discussion), they may want to re-emphasize whether the positive mosquito pools used in their analyses were WNV-1 or WNV-2 - both circulate in this region of Italy...

Thank you for pointing this out, we appreciate the help to clarify our intended meaning. We have replaced "NY99 strain" with "North American WNV lineage 1" as suggested.

Our discussion was intended to emphasize differences in geographic and ecological context rather than lineage-specific biological differences. Specifically, we wanted to highlight that results derived from studies using North American WNV strains and North American bird species may not be directly generalizable to European ecological settings, regardless of lineage. We did not intend to investigate or comment on any potential differences in biodiversity effects across WNV lineages, as this is beyond the scope of our study and we lack the data to test such hypotheses.

We have revised the relevant discussion section to clarify this point and avoid potential confusion (page 15, lines 487-490):

*“While laboratory studies have demonstrated that many passerine species develop high viremia levels following WNV infection, these findings are largely based on North American species and the North American WNV lineage 1 [68], which may not be generalizable to other geographic contexts [69].”*

**Summary and General Comments**

In this publication, the authors investigate avian biodiversity relative to the presence and relative intensity of WNV circulation. To their advantage, and how this manuscript excels over many others investigating the relationship between biodiversity and pathogen abundance, the authors have a clever sampling design, with excellent scale over a large study area. The search for dilution/amplification effect is never a straight-forward endeavour, and there are many examples of disagreement that stem for differences in methodology. The authors discuss these, and provide adequate discussion to put their analysis within context. However, in general, I think they could do a bit more to justify their use of the models, simply because I like their approach and I wonder how it could be applied to other arbovirus systems.

To summarise their manuscript: For the first part of their study, they categorize their sampling sites according to “WNV intensity” (years of observed positives) in the mosquito population. They then applied these categories to the data from avian monitoring stations to derive a aggregated diversity index per category. They correctly chose to apply multiple estimations of diversity, including rarefaction methods and Chao2 derivations. As they have evidence of a dilution effect, on a rather large temporal scale, they then investigated the relationship between the proportion of positive pools per trap-week, landcover, meteorological parameters, and species richness (divided into passerine and non-passerine species) in a model that accounts for spatial relationships, and incorporates temporal relationships via lagged variables.

The manuscript was really nice to read – the topic is excellent, the approach is well-designed, and the methods are well-established. I admit I have no experience with Bayesian spatiotemporal regression or the INLA-SPDE; however, I have enough background to understand the procedure. The authors did well to devote some of their discussion to the reasoning, benefits, and limitations behind their analyses; and probably the most important sentence from the manuscript “it remains challenging to disentangle whether this reflects a genuine amplifying role…” etc.

Thank you for the thoughtful and comprehensive review. We greatly appreciate the recognition of our study design and methodology, as well as the constructive suggestions for strengthening the manuscript.

My main criticism, is that the authors could add a bit more justification of the spatiotemporal analysis, and the inferences they draw from this. I assume this represents a new application of the INLA-SPDE method, and they should do everything they can to convince us that it can handle the nuances of an arbovirus-vector-host relationship. Namely, I think the assumption that mosquito WNV circulation is a continuous spatially-correlated process across the region needs qualification. I’m not disagreeing, but the evidence for this is indirect and should be provided.

Thank you for pointing this out. We agree that additional justification for the INLA-SPDE method would be useful. We have expanded the methods section to describe why INLA-SPDE is particularly well-suited for this analysis and now cite a recently published study applying the same analytical framework to mosquito surveillance data in Emilia-Romagna, which provides additional methodological context. We also justify the assumption of the continuous spatially correlated process through spatial autocorrelation analysis as follows (page 9, lines 292-305):

*“A Bayesian spatiotemporal model [58] was adapted to examine the relationships between WNV transmission risk and bird species richness, adjusting for environmental covariates in a seasonal context using the Integrated Nested Laplace Approximation - Stochastic Partial Differential Equation (INLA-SPDE) approach implemented in R-INLA (www.r-inla.org) [59, 60]. This approach was chosen because it efficiently models spatially structured ecological processes from irregularly spaced sampling locations, as in the mosquito surveillance network analysed.*

*WNV transmission risk, as measured by VI, was assumed to be a continuous, spatially correlated process across the region. This assumption was validated through spatial autocorrelation analysis, which revealed significant clustering of VI (see S3 Text). The spatial process was represented using a discretized Gaussian Markov random field (GMRF) on a triangulated mesh [61, 62]. The model combines the spatial field (a random effect) with the avian, meteorological, and land use variables (fixed effect covariates) to reconstruct the observed patterns in mosquito WNV circulation.”*

It relates specifically to supporting the justification for using “years of activity” as a measure of “WNV intensity”. For example, the largest proportion of sites had three years activity, and those three years – specifically – represented the most intense years of activity across Europe (increased human cases, increased horse cases, increase detections in birds and mosquitoes). It seems contradictory, but is easily explained and the authors should explain it. It is a point worth stressing, because this manuscript could be foundational for other spatiotemporal approaches to understand arbovirus transmission, where vector(s)-vertebrate host(s) relationships are similarly nuanced.

Thank you for this suggestion. We performed a Pearson correlation analysis between WNV-detection years and the proportion of positive mosquito pools to support its use, which we've added in the method section (page 5, lines 184-188):

*“WNV activity at each trap was quantified as the number of years with at least one mosquito pool testing positive for WNV. This metric was selected because it showed a strong positive correlation with the proportion of mosquito pools testing positive during the study period (Pearson’s r = 0.77, 95% CI: 0.65–0.85, p < 0.001), suggesting that WNV detection frequency serves as a reasonable indicator of local WNV circulation intensity.”*

**Reviewer #5: (The email included an attached Word document containing comments for each section. Since all fields for Reviewer #5 were empty in the email, we assume that their full feedback was provided in the attached document.)**

The manuscript entitled “Association of avian biodiversity and West Nile virus circulation in mosquitoes in Emilia-Romagna, Italy” is a very interesting study as it describes the zooprophylactic effect of avian biodiversity on West Nile virus circulation in mosquitoes in endemic foci located in Northern Italy. Several points mainly must be addressed before considering this manuscript for publication. Thus, I recommend that the authors should address the following comments before considering this manuscript for publication.

Thank you for the constructive feedback on our manuscript. We appreciate the recognition that this study provides valuable insights into the zooprophylactic effects of avian biodiversity on West Nile virus circulation in Northern Italy. We have carefully considered each comment and provide detailed responses below, along with the corresponding revisions made to the manuscript.

**Introduction**

The DEH has been demonstrated in multiple vector-borne zoonotic diseases systems such … Please include the following article:

Chelbi, I. B. Kaabi, M. Derbali, S. Ben Hadj Ahmed, K. Dellagi, and E. Zhioua. 2008. Zooprophylaxis: Impact of breeding rabbits in man-made underground holes around houses on reducing the indoor density of Phlebotomus papatasi, principal vector of Leishmania major, etiologic agent of zoonotic cutaneous leishmaniasis in the Old World. Vector-Borne and Zoonotic Diseases, 8: 741-747.

Thank you for this suggestion. We agree that this paper enriches the background by demonstrating that dilution effects have been discovered in multiple vector-borne zoonotic disease systems. We have added the reference and included cutaneous leishmaniasis as an example in the background section as follows (page 4, lines 110-112):

*“Over the past two decades, the DEH has been demonstrated in multiple vector-borne zoonotic disease systems, such as Lyme disease [25, 26], tick-borne encephalitis [27], cutaneous leishmaniasis [28], and, more recently, Usutu virus [29].”*

**Materials and methods**

I have one comment regarding the calculation of "Biodiversity indicators": Authors used point-count survey with unlimited distances to infer and compare the occurrence and abundance of bird species. However, such a method provides bird detection data that could not be used in interspecific comparisons because of possible differences in detectability among species. Highly conspicuous species may be more detectable than expected by their abundance, and vice versa. By using the chao estimator, this detectability issue has nicely been taken into account in the estimation of species richness. However, this issue was completely ignored when calculating the Shannon and Simpson diversity indices (both are based on relative abundance data), possibly leading to biased estimates. Therefore, I'm wondering if there is a way to convert detections into abundance data? Are there species-specific correction coefficients that may be used to convert the detection data into number of individuals?

Thank you for raising this methodological comment. We agree that detectability differences among species can introduce bias when calculating abundance-based diversity indices from point-count data with unlimited distances.

Unfortunately, to the best of our knowledge, there is no practical way to convert our detection data into actual abundance data. While species-specific detection probabilities and correction coefficients might exist in the literature for some species, applying them would be unfeasible because detection probabilities are highly site- and context-specific, varying with habitat, weather conditions, observer experience, and time of day. Moreover, we would need validated correction factors for all 107 species in our dataset across multiple years and sites.

We agree that this represents a common limitation in ecology studies using point-count data. We acknowledge that the Shannon and Simpson indices may be biased toward more conspicuous species and have added this limitation to the discussion (page 16, lines 533-535):

*“Additionally, the calculation of Shannon’s and Simpson’s diversity indices from point-count detection data may be biased toward more conspicuous species, as these abundance-based indices do not account for species-specific detectability differences.”*

**Discussion**

The authors should discuss how bird migration from North Africa which is endemic for WNV Northward to Italy impact WNV circulation.

From 2013 to 2023, major outbreak of WNND occurred during 2018 in this foci. The author should discuss the relationship between entomological surveillance data and ornithological data sources during 2018.

We thank the reviewer for this valuable suggestion. We acknowledge the importance of understanding how bird migration from North Africa, where WNV is endemic, may impact northward WNV circulation to Italy.

While we agree this would be a crucial research question, several methodological constraints prevented us from adequately investigating this relationship. Our study period (mid-May to September) primarily captures autumn migration patterns rather than the spring migration from North Africa. In the Emilia-Romagna region, spring migration from North Africa typically peaks in April and early May, with most trans-Saharan migrants having already reached their breeding territories by mid-May. Our sampling period therefore misses the peak influx of potential WNV-carrying birds from endemic areas. While our data better capture the autumn migration period (August- September) when birds are moving southward, this is not relevant for understanding how WNV might be initially introduced from North African endemic areas into Italian circulation. Additionally, according to local ornithologists, many common species in our study site include both resident breeding populations and migratory populations. With eBird presence data alone, it is difficult to reconstruct actual migratory patterns and investigate this question.

We did perform spatiotemporal analysis examining the relationship between purely migratory bird species richness and WNV circulation using eBird data. Our results showed a negative association between the richness of purely migratory species (as categorized by ornithologists) at a three-week lag and WNV circulation, which is consistent with the DEH. However, given the aforementioned limitations, we cannot draw conclusions about the specific role of bird migration in WNV circulation.

Regarding the suggestion to investigate the association between entomological surveillance data and ornithological data during the 2018 outbreak, unfortunately the bird census data do not allow this specific analysis due to a lack of observation records for deriving reliable year-specific biodiversity metrics. Similarly, the eBird data provides seasonal rather than year-specific estimates. We have commented on these limitations in the Discussion section (page 16, lines 536-539):

*“A potential drawback of the eBird data is that abundance estimates are based on the eBird citizen science project which by design does not systematically survey pre-defined locations and hence may introduce bias. Furthermore, these estimates reflect seasonal trends and do not capture interannual variations.”*

In the manuscript, we advocate for additional data collection approaches to address these important questions (page 17, lines 579-584):

*“Finally, our study underscores the importance of coordination between disease surveillance and bird monitoring programs to facilitate targeted data collection for understanding the circulation patterns of WNV and other zoonotic diseases.”*

**Conclusion**

There are conflicting findings regarding the impact of host biodiversity leading either to zooprophylactic or zoopotentiation. Taking into account the size of this study by investigating the effect of avian biodiversity on WNV circulation in Culex mosquito populations by analysing 11 years of entomological surveillance data and two ornithological data sources, the findings provide strong evidence of the DEH of WNV in this endemic focus.

We thank the Reviewer for acknowledging the strengths of our analysis and the richness of 11 years of entomological surveillance data and multiple ornithological data sources. We have highlighted in the text the comprehensiveness of the analysis conducted in this study in the context of existing publications on the dilution or amplification effect (page 17, lines 562-567).

*“**The results presented in this study provide the first empirical evidence supporting the DEH for WNV transmission in a European setting. This analysis represents one of the most comprehensive studies examining avian biodiversity effects on vector-borne zoonosis transmission from 11 years of systematic entomological surveillance and multiple ornithological data sources, which has important implications for public health practice and biodiversity conservation.”*

**Reviewer #6**:

**Methods**

The objectives of the study are clearly stated. The study design should be modified to include certain parameters that have not been considered and that could be relevant to the co-modulation of the probability of circulation and transmission dynamics of WNV. It would be advisable to approach the analysis of the data from the study of the association of bird diversity with a focus on the rate of virus circulation rather than the frequency of virus presence over a series of years. The working hypotheses are well presented and the data analysis approach is appropriate, although it could perhaps be somewhat more robust in the authors' first study. There are no ethical implications in the data used by the authors that need to be considered.

Thank you for the constructive feedback. We agree that our outcome metrics should better reflect the rate of WNV circulation in the Farmland Bird Index data analysis, and that factors such as mosquito abundance, which co‑modulate WNV circulation and transmission risk, should be taken into account. Based on your comments, we have made the following main modifications:

For the Farmland Bird Index analysis, although we could not perform analyses linking trap‑level bird diversity and WNV transmission rate due to data limitations, we show that the number of WNV detection years is strongly correlated with infection rate at each trap over the study period (i.e. the proportion of positive pools among all tested pools). This supports the use of this metric, which we now refer to as “WNV detection frequency,” as suggested.

For the eBird analysis, we changed the outcome indicator to the Vector Index (VI), which integrates infection rate and mosquito abundance and therefore provides a more comprehensive measure of WNV transmission risk.

Further details on these points are provided below in our responses to your specific comments.

**Results**

The results obtained are presented clearly and in detail, as are the tables and figures.

Thank you for the positive feedback.

**Conclusions**

Some of the conclusions are not sufficiently supported by the results due to the lack of robustness of the methodological approach used and the data employed. Some limitations in the data should be taken into account in the interpretation of the authors' findings, and the uncertainty in the findings should be presented in more detail. I believe that the authors should also place more emphasis on the health implications that their results may have, as well as delve deeper into how their results can be translated into actions that can be applied in policy-making to prevent cases of WNV.

Thank you for the valuable suggestions. We have expanded the discussion of limitations and uncertainty for both bird data sources and clarified that our conclusions should be interpreted in light of these constraints, as detailed in our responses to your specific comments below.

We have also more explicitly described the public health significance of our findings and how they can inform practice and policy in the Discussion. The revised text now reads (page 17, 562-576):

*“The results presented in this study provide the first empirical evidence supporting the DEH for WNV transmission in a European setting. This analysis represents one of the most comprehensive studies examining avian biodiversity effects on vector-borne zoonosis transmission from 11 years of systematic entomological surveillance and multiple ornithological data sources, which has important implications for public health practice and biodiversity conservation. Our findings suggest that areas characterised by high passerine species richness or low overall avian biodiversity may experience higher WNV transmission risk. In practice, such areas could be prioritised for intensified mosquito surveillance, enhanced blood‑donor and equine screening, strengthened risk communication, and targeted vector control to help prevent human and animal cases of WNV. The analytical approach developed in this study also highlights the value of generating high-resolution bird diversity and abundance maps, which can help improve our understanding of biodiversity's role in disease dynamics and demonstrate the beneficial role of avian biodiversity conservation in reducing the local long-term risk of WNV circulation in the bird, equids, and human populations.”*

**Summary and General Comments**

General comments

The study by Wang et al. seeks to investigate the relationship between the diversity of local bird communities and the circulation dynamics of West Nile virus using a significant set of data from virus surveillance in Emilia-Romagna. The virus surveillance and monitoring programme provides a significant and highly relevant time series of data to deepen on what modulates the infection rate in mosquitoes and the risk of transmission to humans and animals. The study focuses in a very relevant aspect to better understanding WNV ecology and transmission potential, although my impression is that the scarcity of appropriate data on bird diversity encountered by the authors prevents a comprehensive analysis of its effect on the ecological dynamics of the virus and detracts robustness from the findings for the authors' objective.

Thank you for the valuable feedback. We acknowledge the limitations of the data, which however do not prevent this study from providing meaningful insights into this important ecological question. We have addressed each concern below and have made revisions to further strengthen the manuscript.

On the one hand, the authors use data from a national bird survey to derive annual diversity indicators that they relate (in a manner that may be not robust enough for the diversity of elements that can determine local virus circulation in a given year) to the dynamics of WNV infection in mosquito vectors. However, the approach used does not allow for an accurate estimate of the real role of local bird diversity in the dynamics of the virus because the authors are forced to summarise the circulation of the virus in the number of years in which each trap was positive. This estimate of virus circulation frequency is indicative of the probability of virus presence, but does not adequately inform the actual rate of virus circulation in the mosquito population, which is ultimately the relevant finding. A perhaps more appropriate approach for this purpose would be to relate these diversity indicators to the total number of positive Cx. pipiens pools with respect to the total number analysed for each trap and year and in relation to the abundance of Cx. pipiens.

Thank you for sharing these methodological concerns.

Regarding the suggestion to test associations at the trap-year level, the bird census and mosquito surveillance operate as two independent systems (and our efforts to establish a linkage between them is a strength of this study). Unfortunately, the number of bird observations per trap per year (typically 1–2 observations) is insufficient for computing reliable yearly trap-level biodiversity indices. This is why we aggregated the bird observations based on WNV detection frequency groups, and whilst we acknowledge that this temporal aggregation limits our ability to investigate dynamical associations, we believe that a temporally aggregated analysis of the association between biodiversity and WNV transmission provides a valuable contribution to the published literature in the light of the existing evidence and limitations of the existing data. We have revised the text as follows (page 16, lines 528-533):

*“A key limitation of the Farmland Bird Index data analysis is that it was conducted using aggregated trap data rather than individual trap-level observation records. While this ensured sufficient bird observations to derive biodiversity indices at each WNV detection frequency group, it limited the extent to which we could investigate year-specific associations and account for potential confounders such as meteorological and land-use factors at fine spatial resolution.”*

We have also performed a Pearson's correlation test between WNV detection years and the proportion of mosquito pools testing positive over the study period, and found a strong positive correlation, which shows that WNV detection years serves as a good proxy for the rate of viral circulation. We added this justification in the methods section as follows (page 5, lines 184-188):

*“WNV activity at each trap was quantified as the number of years with at least one mosquito pool testing positive for WNV. This metric shows a strong positive correlation with the proportion of mosquito pools testing positive during the study period (Pearson’s r = 0.77, 95% CI: 0.65–0.85, p < 0.001), suggesting that WNV detection frequency serves as a reasonable indicator of local WNV circulation intensity.”*

On the other hand, eBird data, even with all the potential biases that they may have and that the authors have tried to minimise, offer the probability of obtaining more accurate spatio-temporal relationships between bird diversity and the infection rate of local mosquito populations. The authors carry out a very well-developed analytical approach, but ultimately they must transform these data into indicators of bird species richness that do not inform about the actual diversity of the local bird community, thus missing the opportunity to identify how variations in local bird communities modulate the infection rate and, with it, the risk of virus transmission.

One aspect that the authors do not consider in either of the two analyses is the local abundance value of Cx. pipiens based on trap capture data, and I believe that this value is extremely relevant in order to identify the extent to which the frequency of virus detection in the mosquitoes analysed or the temporal infection rate are indicators of the relative abundance (which could be called relative density) of infected mosquitoes in the population. Changes in this value may be truly relevant for inferring the effects of bird communities on virus transmission dynamics.

Thank you for the constructive feedback. Following this suggestion, we changed the outcome indicator in the spatiotemporal analysis of the eBird data from the proportion of mosquito pools testing positive to the Vector Index (VI). The VI is computed as the infection rate estimated from pooled data and captured mosquito abundance per trap-night, which comprehensively reconstructs the WNV transmission risk posed by local mosquito populations.

We performed the INLA-SPDE analysis using the VI as response, and the results remained consistent for non-passerine species richness, passerine species richness, and fully migratory species richness.

This supports the robustness of our findings that passerine species richness is associated with increased WNV transmission risk in *Culex* populations in Emilia-Romagna, while non-passerine richness is associated with decreased transmission risk.

We have updated the manuscript accordingly and have edited the Methods section as follows (pages 8-9, lines 277-290):

*“Vector Index*

*For the 68 mosquito traps that were active throughout each surveillance season and consistently sampled during calendar weeks 18 to 39 from 2013 to 2023, weekly mosquito surveillance records were aggregated across all years. For each trap and week, we utilised the total number of mosquito pools tested for WNV, the number of WNV-positive pools, their unique pool sizes, and mosquito abundance per trap-night to compute the Vector Index (VI). The VI represents the estimated number of WNV-infected mosquitoes collected per trap-night and is derived from:*$\begin{aligned} VI= N\times P\#\left( 1 \right) \end{aligned}$

*where N refers to the mosquito abundance per trap-night, and P denotes the estimated infection rate [56]. Infection rates were obtained using the PooledInfRate R package [57]. A key advantage of using the VI Vector Index as the response variable for the spatiotemporal analysis is in the integration of vector species infection rate with vector abundance data, which provides a composite measure of WNV transmission risk [56].”*

The authors are cautious in their assertions and conclusions, but the findings are actually less robust than the authors consider them to be in order to understand the real effect of the bird community on the dynamics of virus circulation. I think it is important for the authors to provide more depth on the uncertainty associated with their approach in robustly estimating the association between bird diversity and virus dynamics. I think the study is interesting enough to be published, but I believe the authors should make a more concerted effort to clarify the aspects that may not be as robust, such as whether the association between the annual detection frequency of the virus and bird diversity indices supports a dilution effect on transmission as robustly as the authors assert.

We appreciate the constructive feedback and agree to give more space to the discussion of the limitations of the data and interpretation of the results. We summarise below an extract from the Discussion where we comment on the points raised by the Reviewer (page 16, lines 528-552):

*“A key limitation of the Farmland Bird Index data analysis is that it was conducted using aggregated trap data rather than individual trap-level observation records. While this ensured sufficient bird observations to derive biodiversity indices at each WNV detection frequency group, it limited the extent to which we could investigate year-specific associations and account for potential confounders such as meteorological and land-use factors at fine spatial resolution. Additionally, the calculation of Shannon’s and Simpson’s diversity indices from point-count detection data may be biased toward more conspicuous species, as these abundance-based indices do not account for species-specific detectability differences.*

*A potential drawback of the eBird data is that abundance estimates are based on the eBird citizen science project which by design does not systematically survey pre-defined locations and hence may introduce bias. Furthermore, these estimates reflect seasonal trends and do not capture interannual variations. Additionally, because eBird abundance estimates are relative and standardised within each species, they do not allow for direct comparisons between species, limiting our analysis to species richness rather than more comprehensive biodiversity metrics. As a result, while our findings show a positive association between passerine species richness and VI representing WNV transmission risk, it remains challenging to disentangle whether this reflects a genuine amplifying role or simply their higher availability as blood-meal sources, considering that Passeriformes is the largest avian order. Another limitation of the study is in the lack of sufficient data to identify which specific passerine groups or species may be responsible for any potential amplifying effect. These limitations are common to most bird data sources, given the difficulty of accounting for heterogeneity in detection and reporting rates across species. Finally, although we adjusted for environmental covariates that might affect WNV transmission risk, we could not incorporate ecological confounders such as the abundance of birds or mammals that sustain Culex populations over the season, due to the unavailability of data with sufficient temporal resolution for our study site.”*

The finding of a relationship between passerine and non-passerine richness and the rate of virus circulation is interesting and supports previous results, but it is not a novel finding based on the state of the art of knowledge of the ability of different groups of birds to replicate WNV. Furthermore, it does not provide evidence of which groups within local passerine bird communities may be relevant to the dynamics of virus transmission. It could be that there is a positive correlation between the detectability of passerine species and their abundance that masks an effect that is actually associated with an increase in particular passerine species. A good year from a trophic resource point of view could increase the local attraction of passerines while significantly increasing the abundance of key resident birds hosting Cx. pipiens and WNV.

Thank you for sharing this comment. As shown in Figure 4 in the manuscript, most of the local birds in our study sites are non-passerine species, with relatively fewer passerine species present. As a result, if we were to examine specific families or groups of passerine birds, the spatiotemporal variation in species richness would be insufficient to detect meaningful associations. We have incorporated this point in the limitations of the eBird analysis as follows (page 16, lines 539-549):

*“Additionally, because eBird abundance estimates are relative and standardised within each species, they do not allow for direct comparisons between species, limiting our analysis to species richness rather than more comprehensive biodiversity metrics. As a result, while our findings show a positive association between passerine species richness and VI representing WNV transmission risk, it remains challenging to disentangle whether this reflects a genuine amplifying role or simply their higher availability as blood-meal sources, considering that Passeriformes is the largest avian order. Another limitation of the study is in the lack of sufficient data to identify which specific passerine groups or species may be responsible for any potential amplifying effect. These limitations are common to most bird data sources, given the difficulty of accounting for heterogeneity in detection and reporting rates across species.”*

The authors consider several environmental covariates to study the spatiotemporal association between bird richness and WNV circulation rate, but these covariates do not include any related to the hosts that sustain Cx. pipiens. No variables are included for the abundance of birds or any mammals that could sustain the Cx. pipiens population, even though at the spatial scale of the study, host availability is expected to be one of the most important factors in the population dynamics of a haematophagous arthropod. I believe it is important to explain and justify why these types of covariates are not considered.

Thank you for raising this point. We agree that the abundance of birds and mammals sustaining mosquito populations represents a critical ecological factor. Unfortunately, to the best of our knowledge, comprehensive host abundance data are not available for our study region at intra-annual resolution. We have highlighted this important limitation in the discussion as follows (page 16, lines 549-552):

*“Finally, although we adjusted for environmental covariates that might affect WNV transmission risk, we could not incorporate ecological confounders such as the abundance of birds or mammals that sustain Culex populations over the season, due to the unavailability of data with sufficient temporal resolution for our study site.”*

Specific comments
It is unclear whether the data used for the period 2013–2023 comes solely from light traps baited with CO₂ or also from gravid traps for the period 2013–August 2017.

Thank you for this question. We have clarified the data sources in the methods section as follows (page 5, lines 158-162):

*“The mosquito data used in this study were obtained from 68 traps that were active throughout 2013-2023, the vast majority of which were CO_2_-baited traps. 14 gravid traps were deployed only during the first five seasons (2013-2017) and were subsequently replaced by CO₂-baited traps at the same locations. Collections from gravid traps accounted for 8.6% of all mosquito pool entries in the dataset.”*

Statistical analysis of Farmland Bird index. The authors refer to the parameter estimated to measure the frequency or persistence of WNV circulation as circulation intensity, but given that it is a frequency of detection of the virus's presence in each year, it is not a true intensity value because it does not estimate the number of positive pools in each year, but only the presence/absence of virus detection in each year and each capture area. I suggest that they use the term WNV detection frequency.

Thank you for this suggestion. As mentioned above, a correlation analysis shows a strong positive correlation between the proportion of WNV-positive pools and WNV detection years across the study period, which justifies the use of WNV detection years as an indicator for WNV activity. We agree that "intensity" is not the most accurate term and have changed the terminology to "WNV detection frequency" throughout the text and figures, as suggested.

I cannot see whether total bird richness was associated with the probability of detecting WNV-positive pools when this parameter is actually more important for assessing the effect of the local bird community as a whole compared to passerine or non-passerine richness. Was the parameter excluded from the selected model? I believe it would enrich the discussion to indicate the role of this global parameter as a joint indicator of richness in the local bird community.

Thank you for raising this important point. You are correct that total species richness metrics were not retained during variable selection. Across all model series, passerine and non-passerine species richness were consistently selected in favour of total species richness. We conducted a sensitivity analysis using only total species richness metrics alongside the same set of environmental covariates. In this analysis, total species richness at a three-week lag was retained but showed no statistical association with VI representing WNV transmission risk, as the 95% credible interval crossed zero.

We included this additional analysis as a sensitivity analysis in the Methods section (page 10, 338-340):

*“We also performed a sensitivity analysis using the same analytical framework but only including total species richness metrics to investigate the potential joint effect of the local bird community, without distinguishing passerine and non-passerine richness variables.”*

The results are presented in S4 Table and described in the Results section (page 14, lines 438-441):

*“In the sensitivity analysis investigating the role of the overall richness of the bird community in WNV transmission risk, total species richness was not significantly associated with VI, as the 95% credible interval (CI) crossed zero (mean coefficient: 0.000, 95% CI: [-0.001, 0.001]; S4 Table).”*

We have expanded the discussion highlighting the importance of subdividing host communities when testing the dilution or amplificant effect hypotheses. Given that non-passerine species richness was negatively associated with WNV transmission risk while passerine richness was positively associated, but no association was observed for overall species richness, this indicates that amplifying effects from competent hosts may counteract dilution effects from incompetent hosts.  We have added this finding to the discussion as follows (page 15, lines 503-509):

*“While we observed that non-passerine richness was negatively associated and passerine richness was positively associated with VI, in the eBird analysis we found no association between total bird species richness and VI, suggesting that total species richness alone may be insufficient for assessing biodiversity-disease relationships, as amplifying and dilution effects from hosts with different competence levels may counteract each other. Our results highlight the importance of accounting for community composition and of considering species-specific richness metrics when investigating associations with WNV risk estimates.”*
