# Supplementary material for: Association of avian biodiversity and West Nile Virus circulation in Culex mosquitoes in Emilia-Romagna, Italy
Source: PLoS Negl Trop Dis. 2026 Mar 6;20(3):e0014076. doi: 10.1371/journal.pntd.0014076 (PMC12978567; doi:10.1371/journal.pntd.0014076)
Supplement: Attachment — Submitted filename: Response_to_Reviewers_auresp_2.docx [file pntd.0014076.s013.docx]

**Association of avian biodiversity and West Nile Virus circulation in Culex mosquitoes in Emilia-Romagna, Italy**

**Point-by-point response letter**

We would like to thank the Reviewers for their valuable feedback. Below, the reviewer comments are presented in black and numbered sequentially. Our responses are provided in blue, and quoted text from the manuscript appears in italics.

**Reviewer #1:**

Summary and General Comments

The comments raised and suggestions proposed at my initial review have been addressed adequately at the revised manuscript.

We thank the Reviewer for the positive feedback.

**Reviewer #3:**

Summary and General Comments

The authors have improved the manuscript according to my suggestions. I think it is a very fine contribution to the understanding of WNV ecology in Europe, and a test of the dilution hypothesis in general. However, I still take issue with one minor point that the authors have not adequately addressed.

From their reply and modification, I understand that they did not have the data to support a test of the effect of WNV lineage on their model. They have acknowledged a comparison with studies in North America, where WNV-1 circulates, in the discussion ("these findings are largely based on North American species and the North American WNV lineage 1 [68], which may not be generalizable to other geographic contexts [69]") and state in their reply "We did not intend to investigate or comment on any potential differences in biodiversity effects across WNV lineages".

However, they must state - explicitly in the manuscript - whether their dataset discriminated between WNV lineages, and note the potential limitations of not differentiating between lineages in their analysis. Both lineages co-circulated in the region, during the time of the study. Phenotypic differences between these viral lineages (in terms of host/vector competences) may obfuscate their findings, and this limitation should be explicitly acknowledged - also potentially for readers who might not be aware that this was the case!

Thank you for recognising the contribution of our study and for this constructive comment. We agree that the potential influence of different WNV lineages on our findings is not neglectable and should be explicitly acknowledged in the manuscript. Lineage was assessed for all mosquito pools at each trap. These data show that lineage 1-positive pools are very limited and concentrated only towards the end of the study period. In 2022-2023, we detected 150 pools positive for lineage 2, compared to only 9 pools positive for lineage 1 and 3 pools positive for both lineages. We have added this information to the Results section (page 10, lines 356-359):

*“We considered all WNV-positive mosquito pools irrespective of lineage, despite WNV lineage 2 was predominant during the study period (i.e. only 9 pools were positive for lineage 1 and only 3 pools were positive for both lineage 1 and 2 during 2022–2023, compared to 150 pools that were positive only for lineage 2).”*

We have also highlighted in the Discussion that the dilution effect observed is mostly based on WNV lineage 2 and may not be directly applicable to lineage 1 (pages 16-17, lines 558-564):

*“It is also important to note that, as WNV lineage 2 was largely dominant in Emilia-Romagna during the study period according to mosquito surveillance, our results should be interpreted primarily as reflecting the association between avian biodiversity and the transmission dynamics of WNV lineage 2 in this region. They may not be directly generalisable to lineage 1 or to settings where lineage 1 predominates, given potential phenotypic differences between lineages, for instance in avian reservoir competence or in the vector competence of local Culex populations.”*

**Reviewer #6:**

Methods

The authors have considered my recommendations of taking into account vector abundance on WNV transmission indices and other methodological considerations.

Results

The authors have demonstrated the robustness of their findings, which was one of my main concerns, and clearly stated it in the revised manuscript.

Conclusions

Yes and limitations are clearly presented for a correct interpretation of the conclusions by readers.

We thank the Reviewer for the positive feedback and for their helpful suggestions, which have contributed to strengthening the methodological robustness and interpretability of our study.
